# Improving access to primary health care: a cross-case comparison based on an a priori program theory

**DOI:** 10.1186/s12939-021-01508-0

**Published:** 2021-10-11

**Authors:** Catherine Spooner, Virginia Lewis, Cathie Scott, Simone Dahrouge, Jeannie Haggerty, Grant Russell, Jean-Frederic Levesque, Emilie Dionne, Nigel Stocks, Mark F. Harris, IMPACT team

**Affiliations:** 1 Centre for Primary Health Care and Equity, University of New South Wales, Sydney, New South Wales Australia; 2 LaTrobe University, Melbourne, Victoria Australia; 3 University of Calgary, Calgary, Alberta Canada; 4 C.T. Lamont Primary Health Care Research Centre, Elisabeth Bruyère Research Institute, Ottawa, Ontario Canada; 5 Department of Family Medicine, McGill University, Montreal, Quebec Canada; 6 Department of General Practice, Monash University, Notting Hill, Victoria Australia; 7 Agency for Clinical Innovation, NSW Health, Sydney, New South Wales Australia; 8 St. Mary’s Research Centre, McGill University, Montreal, Quebec Canada; 9 Department of General Practice, University of Adelaide, Adelaide, South Australia Australia

**Keywords:** Primary health care, Equity, Access, Vulnerable populations, Theory-based evaluation

## Abstract

**Background:**

Inequitable access to primary health care (PHC) remains a problem for most western countries. Failure to scale up effective interventions has been due, in part, to a failure to share the logic and essential elements of successful programs. The aim of this paper is to describe what we learned about improving access to PHC for vulnerable groups across multiple sites through use of a common theory-based program logic model and a common evaluation approach. This was the IMPACT initiative.

**Methods:**

IMPACT’s evaluation used a mixed methods design with longitudinal (pre and post) analysis of six interventions. The analysis for this paper included four of the six sites that met study criteria. These sites were located in Canada (Alberta, Quebec and Ontario) and Australia (New South Wales). Using the overarching logic model, unexpected findings were reviewed, and alternative explanations were considered to understand how the mechanisms of each intervention may have contributed to results.

**Results:**

Each site addressed their local access problem with different strategies and from different starting points. All sites observed changes in patient abilities to access PHC and provider access capabilities. The combination of intended and observed consequences for consumers and providers was different at each site, but all sites achieved change in both consumer ability and provider capability, even in interventions where there was no activity targeting provider behaviors.

**Discussion:**

The model helped to identify, explore and synthesize intended and unintended consequences of four interventions that appeared to have more differences than similarities. Similar outcomes for different interventions and multiple impacts of each intervention on abilities were observed, implying complex causal pathways.

**Conclusions:**

All the interventions were a low-cost incremental attempt to address unmet health care needs of vulnerable populations. Change is possible; sustaining change may be more challenging. Access to PHC requires attention to both patient abilities and provider characteristics. The logic model proved to be a valuable heuristic tool for defining the objectives of the interventions, evaluating their impacts, and learning from the comparison of ‘cases’.

## Background

For more than three decades, health care reforms have attempted to address health inequities by improving access to primary health care (PHC) for vulnerable populations in most Western countries [1]. Despite this focus, significant barriers to access remain and vulnerable populations continue to experience inequitable access to needed services [2]. Given the resources that have been directed at addressing these issues, why do such inequities remain?

While promising reforms to address access inequity have emerged, the lack of widespread adoption of innovations lies partially in a failure to effectively articulate, share and embed the underlying logic and mechanisms of effective interventions across many contexts [3]. Theory-based implementation and evaluation approaches (e.g. program logic, theory of change, realist evaluation) provide one method for more clearly understanding the essential elements of programs and their rationale [4].

Despite the lack of progress, there continues to be widespread recognition of the value of scaling and spreading interventions that have achieved desired outcomes in some contexts and hold promise for addressing pressing health and social issues across other contexts [3, 5]. While support for scale and spread through evidence-informed implementation is pervasive, many questions remain about how to do this successfully, particularly when implementing interventions in complex health systems across diverse contexts [6, 7].

In this paper, we describe how a theory-based approach, using program logic to illustrate the expected changes and causal pathways, guided the implementation and evaluation of interventions across six contexts. The knowledge gained from this program of research contributes to the evidence needed to support the scaling and spreading of interventions.

Innovative Models Promoting Access-to-Care Transformation (IMPACT) was a five-year research program through which PHC innovations were designed, implemented, and evaluated. The focus of all interventions was to improve access to appropriate PHC for vulnerable populations. A common approach to design, implementation, and evaluation was used across six contexts in three Canadian provinces (Alberta, Ontario, Quebec) and three Australian states (New South Wales (NSW), South Australia, Victoria). In each region, decision makers, researchers, clinicians and, in some contexts, community members, formed Local Innovation Partnerships (LIPs) to support design and implementation of the intervention and guide local research activities [8]. There were some aspects of the context that were shared across LIPs at national levels (e.g. systems for payment of general practitioners/family physicians (GP/FP), roles of GPs/FPs in the health system, and overall standards of care provided), but there were differences between countries, provinces/states and at the local level. For example, patients do not register with practices in Australia, while each province in Canada takes a different approach to allocating patients to FPs. Such differences between settings added to the complexity of conducting a coherent research program. While local context and population characteristics influenced intervention designs, they are not the focus of this paper.

Levesque et al.’s conceptual framework of access to health care informed the design, implementation and evaluation components of this research (e.g. framing priority areas of focus within each of the participating communities and providing an initial organizing framework for the program logic) [9]. The Levesque et al. conceptualization described both demand- (patient) and supply-side (provider) factors that influence access to health care and suggested strategies to address access throughout the process of obtaining care. Demand-side factors were patient abilities to perceive, seek, reach, pay for and engage with health care. Supply-side factors were the approachability, acceptability, availability, affordability, and appropriateness of health services.

The aim of this paper is to describe what we learned about improving access to PHC for vulnerable groups across multiple sites through use of a common theory-based program logic and a common evaluation approach.

## Methods

A full description of the IMPACT study is published elsewhere [8]. The IMPACT evaluation used a convergent mixed-methods design involving longitudinal (pre and post) analysis of the six interventions. We adopted a theory-based evaluation approach to guide the evaluation of the IMPACT initiative. This involved developing a representation (logic map) that focused on the overarching initiative, capturing the intended consequences of individual projects as part of the whole, without expecting any individual program or component to necessarily achieve all the stated goals. This was done at a face-to-face meeting of all investigators at an early stage in the study.

The overarching logic map incorporated the domains of Levesque et al.’s conceptualization of access to PHC [9]. Hypotheses were generated at the level of each LIP about the intended consequences of their interventions. This represented constructs at a high level, and each LIP identified how their intervention mapped to the overarching program logic as they were developing and refining the intervention. In this, the inputs/activities were not articulated in detail; each LIP further elaborated the program logic to represent the inputs/activities for their intervention. This representation was also the framework for synthesis across the six sites, including guiding data collection using common measures and analysis of findings.

Study participants included vulnerable populations (people who experienced barriers to accessing PHC), PHC practices, their clinicians and administrative staff, service providers in other health or social service organisations, intervention staff and members of the LIP teams, including consumers and policy advisors.

Data for the whole study were to be collected prior to or at commencement and 3–6 months after the interventions. Assessment of consequences of the intervention activities for individuals and organisations relied on surveys and semi-structured interviews (and, in some settings, direct observation) of participating patients, providers and PHC practices.

Ethics approval for the evaluation of the interventions was granted for each site and overall approval from St Mary’s Hospital (Montreal) SMHC #13–30.

### Sample

Criteria for inclusion in this analysis were:

- The intervention targeted the PHC setting.
- The site had sufficient patient-level post-intervention data for the hypothesized consequences to be assessed.

Two of the study sites did not meet these criteria (Victoria, South Australia), leaving four sites for this analysis: NSW, Alberta, Ontario, and Quebec.

### Data

While the general principle of pre-post data collection was maintained, differences in the nature and duration of the interventions meant that the actual timelines for data collection varied (Table 1).

**Table 1:** Duration of intervention and data collection relevant to this paper in each site

	NSW	Alberta	Ontario	Quebec
**Intervention**				
Contact with patients	Initial health check appointment with PN Patient-initiated contact with website 6-week follow-up with nurse	1 day “pop-up” event – duration of contact determined by patient need and engagement.	Patient meets navigator – once or more frequently with or without phone follow-up	Single phone call (some received a follow-up call)
Contact with practice/ organisations	Clinical audits to identify clients One visit pre and two visits during intervention	None as part of the intervention	Displaying information about community services and navigator	None as part of the intervention
Contact with providers	Self-guided training video pre intervention Feedback on clinical. audit	One-off or repeated participation in up to 7 pop-ups	Information about community services Referral practice change	None as part of the intervention
**Patient-level data**				
Baseline survey	Prior to health check	None	Prior to meeting navigator	Before or just after first phone call
Baseline qualitative interview with a subsample	N.A.	N.A.	N.A.	Before or just after first phone call
Post intervention survey	6 months after intervention started	On the day, at end of engagement with pop-up	3 months after intervention ended	3 months after first call
Post intervention qualitative interview with a subsample	6 months after intervention started	3–6 months after the intervention	1–3 months after post survey	3 months after first call
**Provider-level data**				
Baseline survey	Following practice consent to participate	N.A.	Following practice consent to participate	Following practice consent to participate
Post intervention survey	9 months after intervention started	3–6 months after participation in each pop-up	1 month prior to end of intervention	9–16 months after pre-intervention survey (6 to 9 months after intervention start)
Post intervention qualitative interview with a subsample	9 months after intervention started with a subset of GPs and PNs	After each pop-up	1–3 months after post-survey	N.A.
**Practice/ organization-level data**				
Baseline survey	Following practice consent to participate	N.A.	Survey following practice consent to participate	Survey following practice consent to participate
Post intervention survey	9 months after intervention started	N.A.	1 month prior to end of intervention	9–16 months after pre-intervention survey (6–9 months after intervention start)

Data came from multiple sources via the data collection methods described above with a focus on the key domains described in the program logic, including:

- Data on vulnerable patients’ abilities to access care (perceive, seek, reach, pay, engage) came from interviews.
- Providers’ confidence/knowledge to support vulnerable patients was assessed through surveys and interviews.
- Organizational processes/policies to support vulnerable patients were assessed through organizational surveys and interviews with key stakeholders.

The hypothesized pathways within the logic model that represented the intended consequences for each intervention provided a guide to analyzing the relationships between elements of the model. Consideration included developing hypotheses about the mechanisms by which each intervention might have contributed to an observed change in the measured constructs, and the relationships between them. This paper uses the term “*consequences*” to describe the changes for patients, providers and organizations that were expected to follow from the activities of the interventions and their immediate effects, or through the causal relationships between different domains described in the logic models.

### Analysis

The analysis approach was based on evaluation methods described by The Kellogg Foundation and Goodrick [10, 11] and informed by Crabtree et al.’s approach to meta-synthesizing results [12]. Data analysis included exploring key patterns in qualitative and quantitative data to identify differences between observed/expected consequences and between cases. Using the overarching logic model unexpected findings were reviewed and alternative explanations were considered to understand mechanisms of each intervention that may have contributed to results. This was consistent with Goodrick’s recommended cross-case comparison approach [10]. In some cases, this process involved non-researcher members of the LIP, while in other cases it was undertaken by key members of the research team. The potential contribution of different characteristics of the interventions and/or the contexts in which they were implemented were considered as part of the analysis. Described as “qualitative comparison analysis” this approach used qualitative and quantitative data to “focus on the relationships among combinations of potential causal conditions within and across cases” (p7) [10].

## Results

**Fig. 1:**
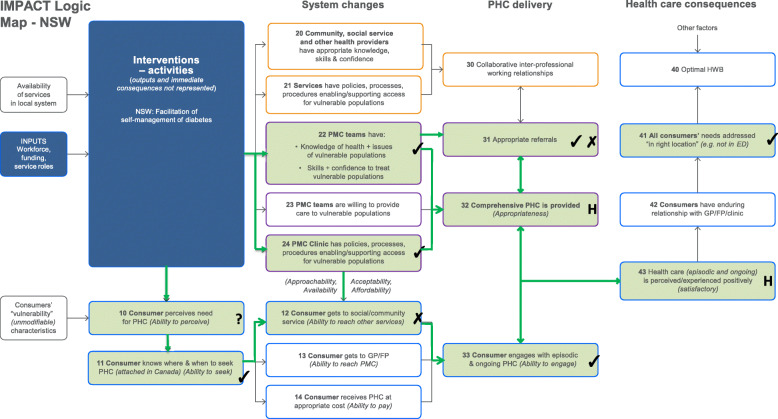
NSW logic model

**Fig. 2:**
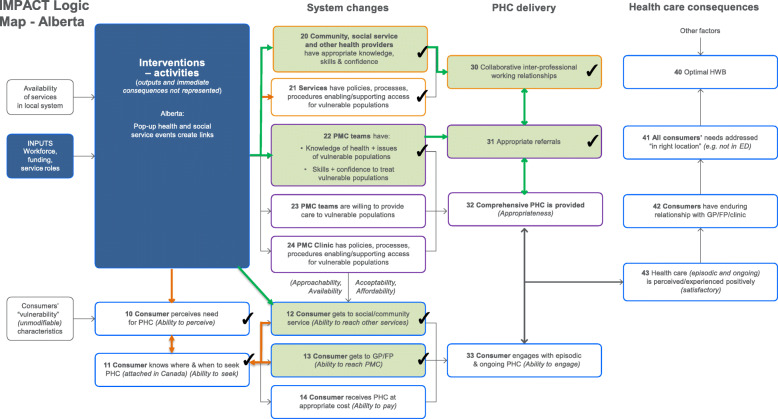
Alberta logic model

**Fig. 3:**
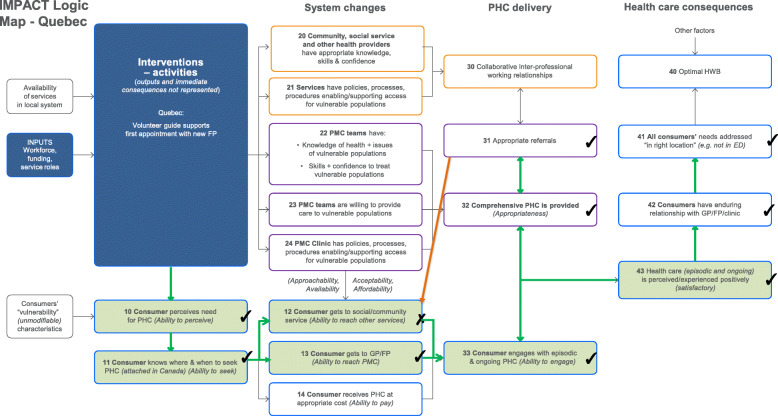
Quebec logic model

**Fig. 4:**
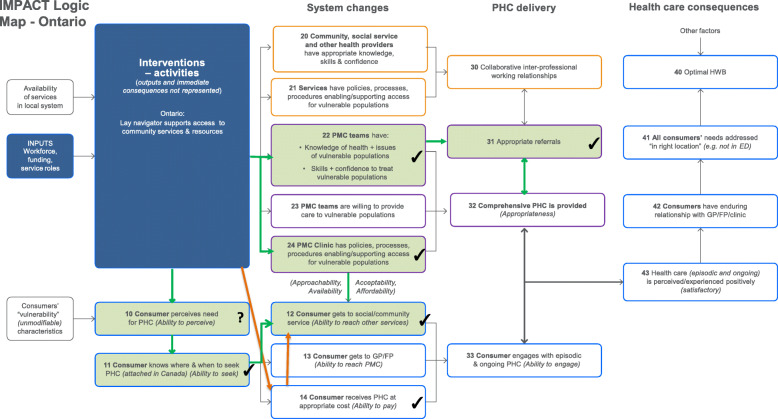
Ontario logic model

**Fig. 5:**
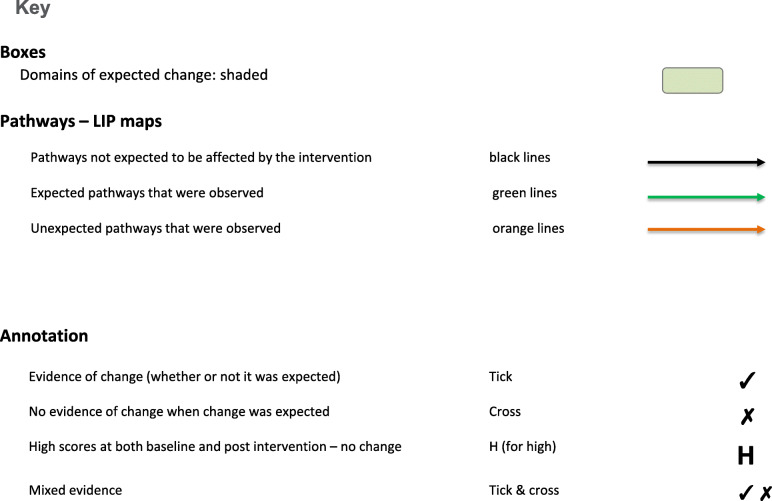
Key to logic models

The results are presented via text (for description), figures (for illustration) and tables (to provide key evidence). Each intervention is described below with reference to the way it maps against the program logic models (PLMs) in Figs. 1, 2, 3, and 4. The key to the Figures 1-5 is in Fig. 5. The numbers in parentheses in the descriptions refer to the box numbers in the PLMs, and describe the key constructs captured in the PLM. Key evidence from different data sources on whether consequences were or were not observed are presented in Tables 2, 3, 4 and 5. Individual projects have or will report more detailed results of their evaluations. Results are based upon unpublished and published reports.

**Table 2:** Evidence of effects in NSW

Box	Impact	Evidence	Source
10	Consumer ability to perceive need	Not measured.	
11	Consumer ability to seek	Significant improvement on score on ‘Ability to seek’ (Scale; 1 = Not easy at all to 4 = Very easy) increased from 3.2 to 3.4 *p* = 0.006.	Patient surveys
12	Consumer ability to reach social/ community services	There were no significant changes in responses to the question: In the last 6 months, have you used a health or social service in the community for a specific health problem?	Patient surveys
22	GP knowledge, skills, confidence	Significant improvement in scores out of 10 for confidence in providing care to patients with poorly managed diabetes from 7.99 to 9.27 (*p* = 0.03).	Provider surveys
24	Clinic policies	9 of 10 practices reported the intervention changed the way the practice organizes access or care for patients with poorly managed Type 2 diabetes: a little (10%), quite a lot (50%) or a great deal (20%). Some clinics had instituted recall systems as part of the study to help with recall of patients for health checks.	Practice surveys
31	Appropriate referrals	GP self-report provided mixed results. Significant increase in the frequency of someone from the clinic helping patients to make the appointment for a referral; *p* = 0.046 No significant changes in frequency of: • providing information on different referral options • allowing patients to choose which referral option suits them • referring patients to self-mgt education.	Provider surveys
32	Appropriate primary care	There was no significant improvement in patient reports that the GP provided everything they needed to help them manage their health. However, there was a ceiling effect with a baseline score of 3.8 out of 4 (4 = yes, definitely).	Patient surveys
33	Consumer ability to engage	Significant improvement in patients’ reports of how easy it was to explain their problems to their health professionals. On a scale of 1 to 4 (1 = Not easy at all; 4 = Very easy), patient scores changed from mean = 3.26 to mean = 3.50 *p* = 0.007.	Patient surveys
		GPs and patients suggested increased engagement of patients, although not universally.	Interviews
41	Consumer needs addressed at right location	Significantly fewer patients reported spending one or more nights in a hospital after the intervention.	Patient surveys
43	Healthcare is perceived /experienced positively	Responses to question: “Did you have confidence and trust in the person you saw or spoke to?” did not change – very high at baseline and follow-up (3.9/4) i.e. ceiling effect.	Patient follow-up survey

**Table 3:** Evidence of effects in Alberta

Box	Impact	Evidence	Source
10	Consumer ability to perceive	Patients’ knowledge about PHC services increased, contributing to increased patients’ ability to perceive need for care.	Interviews
11	Consumer ability to seek	Exposure to services at the pop-ups improved patients’ knowledge about healthcare options they could access after the pop-up.	Interviews
12	Consumer ability to reach social/ community services	Patients were more aware of how to reach services after the pop-up.	Interviews
13	Consumer ability to reach PMC	Primary care practitioners accepted new patients at the pop-ups for ongoing PHC and consumers were able to reach these services after the pop-up.	interviews
20, 22	Health providers (incl GP/FPs), community, social service providers have knowledge skills & confidence	After the pop-ups, service providers reported they understood barriers faced by people accessing care and how to mitigate these barriers. Interview data indicated this knowledge was enhanced by participation in the intervention.	Other provider survey, interviews
21	Services have policies, processes, procedures	Service providers reported they assessed and, where required, modified their organization’s policies, processes and procedures based on what was learned at the pop-up events.	Interviews
30	Collaborative inter-professional working relationships	After the pop-ups, service providers reported that their organizations do inter-organizational planning “well”. Interview data suggested increased collaboration between service providers because of their experience at the pop-ups.	Other provider survey, interviews
31	Appropriate referrals	After the pop-ups, service providers reported that their organizations do referrals “well”. Interview data indicated that service providers continued to implement the principle of warm handoffs in standard care after the pop-ups.	Other provider survey, interviews

**Table 4:** Evidence of effects in Quebec

Box	Impact	Evidence	Source
10	Consumer perceives need	All patients appreciated the telephone outreach, and several patients indicated that they did not realize until after receiving the call that they needed the information that was provided.	Qualitative interviews
11	Consumer ability to seek	Significant increase in score on ‘Ability to seek’ scale (1 = Not easy at all to 4 = Very easy) from 2.8 to 3.3, *p* = 0.000.	Patient survey
12	Consumer gets to social/ community service relative to need	Reported use of community health services not significantly increased from 6% (3/51) at baseline to 17% (*n* = 8/48) at follow-up (*p* = 0.13). Helpfulness of services received not significantly increased from an average of 1.6 (*n* = 14) to 3.3 (*n* = 44) (*p* = 0.020) where 1 = “no, not at all” and 4 = “yes, absolutely”.	Patient Survey
13	Consumer gets to GP/FP	Among patients who tried to contact their provider by telephone: At baseline, 37% reported it was ‘very easy’ compared to 73% post-intervention.	Patient Survey
31	Appropriate referrals	Referral to community health and social services by the usual physician increased significantly from 5% at baseline (1/19 with a usual place of care) to 14% (6/42 reporting on new physician) at follow-up (*p* = 0.06). (NB – providers were different).	Patient survey
32	Appropriate PMC is provided	Patients reported the extent to which their doctor or nurse provided everything needed to manage their health on a 4-point scale from 1 = *not at all* to 4 = *yes, definitely*. Mean scores increased from 3.4 at baseline (*n* = 24, referring to usual source of care) to 3.8 at follow-up (*n* = 54, referring to new FP), *p* = 0.08.	Patient survey
33	Consumer engages with care	Patients’ abilities to explain their problems to health professionals increased from 3.4 at base line (mostly easy) to 3.8 at follow-up (mostly very easy), *p* = 0.002. At follow-up interviews, patients reported that the visit preparation tools had helped them have more effective visits and to better understand their own needs.	Patient survey Qualitative interview
41	Consumer needs addressed at right location	The rate of forgone care because of access difficulties dropped from 41% at baseline to 12% at follow-up. The rate of emergency-room use fell from 20% (12/60) at baseline to 11% at follow-up (6/54, 4 were repeat users).	Patient survey
42	Enduring relationship with GP/FP	Reported attachment to a specific responsible professional increased from 15% (6/60) at baseline to 89% (48/54) at follow-up, *p* < 0.000.	Patient survey
43	Healthcare is perceived /experienced positively	Reports of patient-centered care increased between baseline and follow up: • 26 to 48% reported their provider explored patient concerns about health • 48 to 68% gave importance to patient’s ideas about health • 48 to 85% put patients at ease to discuss issues.	Patient survey

**Table 5:** Evidence of effects in Ontario

Box	Impact	Evidence	Source
10	Consumer ability to perceive need	Not measured.	
11	Ability to seek	Significant improvement on score on ‘Ability to seek’ (Scale; 1 = Not easy at all to 4 = Very easy) increased from 2.7 to 3.0 *p* = 0.000.	Patient survey
12	Consumer gets to social/ community service appropriate to need	52% patients had accessed at least one resource. 55% patients had either accessed the resource, had an upcoming appointment with a health or community service, or were on a waiting list. In total, 57 resources were accessed by study participants, with an average of 1.7 per patient.	Patient survey
14	Ability to Pay	Patients reported the intervention assisted them to access services they could afford.	Qualitative interview
22	GPs/FPs have knowledge and skills	61% of primary care providers reported that the intervention enabled them to refer more to community resources.	Provider survey
24	GP/PC Clinic has policies, processes, procedures enabling/ supporting access	Implemented as part of the study and sustained: Promotional material to help patients be aware of need Referral form embedded in electronic medical record to refer vulnerable patients for navigation.	Observed
31	Appropriate referrals	61% of primary care providers reported that the intervention enabled them to refer more to community resources.	Provider survey

### NSW

#### Population

People with T2D in a disadvantaged urban area attending a general practice (primary medical care (PMC)).

#### Intervention

The NSW LIP implemented a Type 2 diabetes (T2D) intervention at three levels: patient, provider and practice. At the practice level, clinical audits were conducted, reported and discussed with the practice staff. Discussion focused on how reporting of risk factors could be improved. Two subsequent practice visits were conducted to assist the practice to implement the patient intervention, for example, setting up a recall system for patient health checks. At the provider level, training was provided on how to implement the patient intervention (patient recall to health checks). At the provider level, training was provided on how to implement the patient intervention (patient recall to health checks). In addition, provider-level audit reports were given to providers who then reflected upon them as part of their continuing professional development. Patients who had been identified via the audit as having poorly managed T2D and who had agreed to participate in the study were invited to attend a health check at the practice. The health check was based upon the 5As framework [13] and included health assessment, goal setting and an introduction to a web page that provided information on T2D self-management, including referral options. Patients were given a follow-up appointment 6 weeks following referral.

#### Anticipated mechanisms

Through direct contact with providers (PMC teams) and patients, these interventions were intended to increase the assessment and recording of diabetes quality indicators and improve the procedures for recalling patients for health checks (24); to increase provider confidence in supporting diabetes self-management and skills in providing health checks based on the 5As (22); and patient abilities to perceive need (10), seek (11) and reach (12) services outside general practice that provide support for T2D self-management.

#### Intended consequences

These changes were expected to increase provider referrals to other health services that could support patient management of their T2D (31), improve the appropriateness of the care provided to patients (provide more information and support to self-management), (32) to improve engagement with the practice nurse (PN) in relation to diabetes self-management (33) and, as a consequence, the patient experience of health care (43). It was anticipated that, if these outcomes were achieved, the patients’ needs would be more likely to be addressed “in the right location” (not in the emergency department) (41).

#### Observed consequences

There was evidence of a change in practice procedures (24), provider confidence (22), and patients’ ability to seek support for self-management (11). Significant change was not observed in patient ability to reach social/community services (12). This result could have been due to small sample size as non-significant changes were observed. There was some evidence of changes in the pattern of referral (31), but the data were not consistent, and the level of referral was already very high at baseline. Similarly, there was no significant change in patient reports that the GP provided everything they needed to help them manage their health (32), or confidence and trust in their GP (43) because there was no room to move on these as the baseline scores were very high. There was a significant improvement in patient engagement with ongoing primary care (33). There was evidence that significantly fewer patients reported spending one or more nights in a hospital after the intervention (41).

### Alberta

#### Population

Communities with limited access to PHC services.

#### Intervention

A series of ‘pop-up’ health and social service events was delivered in an area with limited access to PHC services. Services at the pop-up events included vaccinations, dental care, mental health services, physician care, health education, service way finding, recreation supports, housing services, and language and literacy services. Pop-ups were held in easy-to-reach locations (I.e., schools, Senior’s Centre, Indigenous Community Centre) on transit routes and transportation support was provided in some instances.

Service providers adopted the role of system navigators by getting to know one another through pre-event rehearsals. Providers greeted people on a personal level before discussing service provision needs using the “How’s Your 5?” conversational tool [14] and plain language descriptions of services. Service providers participated in post-event rapid cycle assessments to improve the approach at subsequent pop-up events.

#### Anticipated mechanisms (during the pop-up)

Through direct contact with patients, the intervention was intended to lead to attendees receiving health, social and community services appropriate to their needs at the pop-up (12), including getting to a GP/FP (13), engaging with the episodic care provided by a range of services at the pop-up, and perceiving it positively.

Through direct participation in one or more pop-ups, the intervention was intended to increase primary health providers’ knowledge of health issues of vulnerable populations and confidence to support people who attended the pop-up (20, 22); and develop positive collaborative interprofessional working relationships (30).

#### Intended consequences (after the pop-ups)

For providers, these changes (20, 22, 30) were expected to be sustained and increase referrals to a broader range of health and social services (31) after the pop-up. For consumers, there was an expectation that ability to reach PMC (13) and social and community services (12) would be higher (sustained increases) after the pop-up.

#### Observed consequences

There was evidence that the increases to knowledge, skills and confidence of health providers (including GP/FPs and community/social service providers) to treat vulnerable populations (20, 22) were sustained after the pop-ups, and that collaborative inter-professional working relationships were sustained (30), leading to appropriate referrals being made after the pop-ups (31). There was evidence that consumers were getting to PMC (13) and other services available in the community (12) after the pop-up, indicating ability to reach was sustained.

#### Unexpected consequences

While some patients were already connected to some community services there was evidence of enhanced ability to seek care after the pop-up (11). People’s awareness of their needs (10) reportedly increased after the pop-ups, perhaps as a consequence of the approach taken to identifying and addressing additional needs (How’s Your 5? and warm hand-offs between providers). Unexpectedly, some service organizations reported they changed organizational policies and processes to enhance service accessibility based on their experiences at the pop-ups (21).

### Quebec

#### Population

People not registered for PMC.

#### Intervention

The Quebec intervention consisted of trained lay volunteer navigators contacting by telephone patients in materially or socially deprived neighborhoods of Montreal prior to a first visit to a newly assigned family physician. The intervention was supported by the regional health organizations that managed a centralized waiting list for family physicians. People could wait on the list for up to 3 years and return to the bottom of the list if they failed to attend when allocated to a family physician. The intervention responded to the low rates of patients in deprived areas presenting to their first visit to register with an assigned family physician. The intervention was implemented at the patient level. There was no provider- or practice-level intervention. The volunteer navigators made multiple attempts to contact the patients to address issues such as any conflict between working schedules and regular clinic business hours. The volunteers explained the importance of attending the first visit, provided access to information about the clinic and supported visit preparation, including preparing documentation and questions. If needed, volunteers provided information about community resources. Volunteers were free to make additional follow-up calls if they thought the person needed more contact (e.g. if they were confused or had questions about instructions received from the practice). In practice most patients received a single call.

#### Anticipated mechanisms

Through direct contact with patients, the intervention was intended to help patients perceive the importance of attending their initial appointment (10) and increase their ability to seek care (11). Information provided to them by the navigator would increase their ability to reach PMC (13) and social/community services (12) and their ability to engage with the episode of healthcare (33).

#### Planned consequences

If the quality of PHC was sufficiently high, it was expected that engagement with PHC would be a positive experience (43).

#### Observed consequences

There was evidence of increased ability to perceive (10) and seek (11), along with ability to reach PMC (13) and ability to engage with care (33). Patients got to the PHC (13) as intended. There was an increase in positive experience of care (43). There was not a significant increase in use of other community health and social services (12) as intended.

#### Unexpected consequences

There was evidence of appropriate direct referrals by their new family physicians (31) and appropriate PHC (32) although this was not directly targeted by the intervention. Patients also reported increased attachment to their FP (42) and reduced use of emergency rooms (41) in line with the proposed program logic.

### Ontario

#### Population

People attending PMC with unmet social- or health-care needs.

#### Intervention

The Ontario intervention was implemented at the practice, provider and patient levels. Lay navigators were trained in patient-centered communication and system navigation. They attended practices to orient providers to the breadth and potential benefits of community resources and encouraged them to direct their patients to these services by writing a “referral form”. Practices were assisted to adapt their existing specialist referral form in the electronic medical record system to allow referral to the navigation services.

Promotional material about the community services available in the region and their potential benefit was displayed in the practices and encouraged patients to discuss their needs for such resources with their provider.

The navigators worked with the patient to prioritize their needs, identify potential barriers to access, and understand their preferences for the type of service that could address their need. They provided informational, emotional, and instrumental support to assist the individual overcome barriers and access the services best suited for their needs and preferences. They also trained the patient to use existing electronic navigation tools. The navigation services ended when the patient was linked to the community service(s), reported being confident finding the service they needed, or no longer wished to receive navigation services. The evaluation took place 3 months post enrolment even if the navigation services had not been completed.

#### Anticipated mechanisms

Through direct contact with providers, the intervention was intended to change PMC providers’ knowledge/skills about referral to community services (22) and increase the referral rates to community resources (31) and to the navigation services for those requiring support (which was facilitated by changes to the electronic medical record referral system (24). At the same time, the promotional material raising awareness about community resources was intended to help the patient perceive previously unrecognized needs (10), and the patient navigator was expected to help individuals overcome access barriers and assist them in reaching the resources they needed.

#### Planned consequences

In the longer term, the patient’s enhanced ability to perceive needs was expected to be reinforced by GPs via the discussion in the course of making referrals to community services (31) and as part of comprehensive PHC provision. The navigation support was expected to enhance the individual’s ability to seek (11) and reach (12) appropriate PHC for their needs.

#### Observed (expected) consequences

There was evidence that the intervention improved practitioner knowledge/skills about referral to community services (22) and clinic referral procedures (24). The majority of providers reported they were more aware of community resources, and more likely to refer to them (31). There was evidence that patients’ ability to seek PHC increased (11) and that the majority reached the social and community services that addressed their needs (12).

#### Unexpected consequences

Many services to which patients were referred required out of pocket payment. We found that navigation supported consumers’ ability to access community services that were free or more affordable (14) and this was a key contributor to them getting the services they needed.

### Cross-case summary of results

#### Strategies used

Although they used a common access framework and mapped their interventions to a shared logic model, each LIP tackled their local access problem with different strategies and from different starting points in terms of level of current engagement with PMC. In Quebec volunteers helped vulnerable unenrolled patients to successfully enroll and attach with a primary care doctor. In Ontario community navigators provided patients already accessing primary care with information to help them access a broader range of services. In NSW PNs conducted a health check that could result in referral and used a purpose-built website to link primary care patients to services and providers to meet their needs more comprehensively. In Alberta multidisciplinary PHC services were provided in underserved communities in a series of one-day “pop-up” events, establishing a new way to access a range of primary healthcare services. In all of these interventions someone spoke to consumers about their needs and linked them directly or indirectly to services they needed.

#### Consequences for patients

Each of the interventions was followed by improvements in patients’ abilities to seek and to reach services. The focus was on getting to social or community services in Alberta as a result of attending the pop-up service and in Ontario as a result of brokered access to other services. In Alberta and Quebec, where the focus was on improving attachment to PMC, patients reported better access to a GP/FP. There were no changes in attachment to PMC in NSW and Ontario because such access was already well-established for those patients recruited and the intervention was an additional service offered to existing patients.

#### Consequences for providers

Changes in primary service organization policies and in provider knowledge, skill and confidence is likely to have been a direct consequence of the training and support provided to practices in NSW and Ontario. In Alberta changes in provider behaviors and service processes were observed as a byproduct of participation in the pop-ups with more provider awareness of vulnerable patients’ needs and how providers could work together to meet them. In Quebec there was no attempt to directly influence primary care practices or providers.

#### Consequences in different sites

The combination of these changes in patient abilities and provider/service capabilities was hypothesized to have influenced how PHC services were provided and how patients engaged with them. This reflects Levesque et al.’s access framework and is consistent with other research [15, 16]. The combination of intended and observed consequences for consumers and providers was different at each site, but the path to consumers receiving care in the right location was the product of delivery of comprehensive PHC and the consumers’ ability to engage. Following the interventions, patients in Quebec reported significant changes in how comprehensively or appropriately their needs were addressed and in NSW patients presented less frequently to hospital.

## Discussion

The development of the program-level logic model was based on assumptions and theory-based hypotheses about mechanisms that would influence consequences for each intervention, and the potential relationships between those consequences. While there was insufficient data to enable statistical analysis of the relationships between variables in the model, the logic model provided predictions that could be tested against the observed changes. The logic model described a complex system, where relationships between domains could be nonlinear and bidirectional. The logic model acknowledges multiple pathways from interventions to provider and patient change. It recognizes and potentially promotes the value of designing interventions that are intended to influence more than one aspect of the complex system via different paths (e.g. patient and provider rather than patient or provider).

Using the model as a synthesizing tool for cross-case comparisons helped to identify and explore intended and unintended consequences of four interventions that appear superficially to have more differences than similarities in ways that would not have been possible if the focus was only on what was expected to happen in each site in isolation. Looking broadly at the impacts of the interventions using a logic model based on Levesque et al.’s conceptualization of access to health care, rather than just looking at the variables that each project thought would be directly influenced, was a strength of the project. This approach facilitated consideration of the connection between elements of the interventions and planned consequences and challenged researchers to consider the similarities and differences across different interventions developed and implemented in different contexts. Having an a priori overarching theory of access to PHC against which to assess different interventions helped to build evidence through synthesis of findings.

One of the key findings of the synthesis across the four interventions was that the causal pathways between the interventions and access appeared to be more complex than anticipated. There was evidence that different interventions had a similar impact on particular abilities. Most of the interventions sought to improve patients’ abilities directly but observed additional and sometimes unexpected flow-on effects between different abilities. This invites consideration of whether there were underlying similarities in the mechanisms operating in each intervention. All of the interventions involved someone proactively reaching out to consumers – through telephone calls and/or face-to-face visits. The effects of this interpersonal contact included the intended positive consequences for patients perceiving a need for the service being offered and reaching promoted care (both community services and primary healthcare).

Another example of this complexity was that one intervention could impact upon multiple abilities in different ways. For example, providing navigation support could improve the ability to seek care, but also to engage with care. This might account for some of the unexpected consequences observed, such as increased ability to pay (Ontario) and ability to perceive (Alberta). In NSW and Quebec, qualitative data suggested that some patients associated the positive experience of the direct contact with their regular (or new) primary care provider, which contributed to a positive experience of PHC. While complexity is not unexpected in intervention research [6], the extent of complexity observed was greater than anticipated. This did not, however, negate the value of the common logic model. In fact, the logic model enabled the complexity to be identified and explored.

### Implications

The sites each developed different approaches to addressing access to PHC that were relevant to their needs and contexts. Despite these differences, most of these interventions were followed by changes to patients’ abilities to seek and engage with care and with providers’ capabilities to provide appropriate care. These occurred despite barriers due to culture, language, health literacy, socioeconomic status, and the complexity of the health sectors. This has implications for wider health reform. Each intervention was a low-cost incremental attempt to address unmet health care needs of vulnerable populations defined by health, social, economic, and geographical disadvantage. They required changes to systems, the way patients related to health services and the routines of health care providers. For example, patients were asked to attend a new preventive check with the PN in NSW and to meet a community health navigator in Ontario.

Based on anecdotal feedback from the LIPs, sustaining these changes has proven to be more challenging. Major structural factors such as the affordability of PHC were not directly addressed, largely because the jurisdictions already had universal health coverage. However even these incremental changes require continuing support to be maintained. Necessary support includes maintaining information systems, informing and educating patients/community members, and funding additional time of volunteer coordinators, CHWs or practices. Although there has been some continuation of the initiatives in three of the four sites, this has been difficult because they are not part of larger health system reforms to improve access or quality of PHC.

The logic model proved to be a valuable heuristic tool for defining the objectives of the interventions and evaluating their impacts. It was based on a common framework described by Levesque et al. One aspect of this framework that was not initially included in the logic model was the interaction between individual patient abilities and provider/service capabilities to produce an enduring relationship with a GP/FP/Clinic. We used the model as a tool to describe the expected patterns of consequences of our interventions and adapted it in response to our emerging findings. During the process of analysis it became clearer that these interact at all levels, not only between the appropriateness of the PHC provided and patients’ ability to engage. The logic model emphasizes that it is insufficient to consider acceptability and availability of services/providers without also considering patient ability to seek or reach and vice versa.

The model also provided a framework for the collaborators in this study to communicate about the different interventions across very different contexts. This enabled sites to develop and validate shared tools for evaluation. It also enabled collaborative teams to learn from each other, share training resources and continue related research. This supported the Monash team to subsequently adapt the Alberta pop-ups in a study of vulnerable populations in Victoria and the UNSW team to develop a program of research on community health navigators in PMC based, in part, on the Ontario study.

### Limitations

There were a number of methodological limitations to the study. The data collection tools did not include ideal measures for the constructs described in the logic model, in part because there were no agreed validated tools available to measure most of the constructs described in the Levesque et al. access framework. In addition, the primary driver in designing the data collection tools was not the logic model developed to represent the IMPACT interventions; some questions were required as part of a mandatory minimum data set by one of the funders. Coupled with the need for intervention-specific questions at most sites, this limited the capacity of the study to add more items that could measure the articulated domains without imposing an excessive respondent burden.

Data collection varied in terms of timing and quantity. While NSW and Ontario included a follow-up survey of participants, there were no measures of longer-term effects or sustainability of consequences.

Given the nature of the research program as a multi-site study with locally relevant innovations, these limitations are not unexpected. These issues support the rationale for taking this program logic approach to synthesis of findings. In the absence of the same intervention implemented in a similar context with a consistent study design, a theory-based approach to synthesis is a pragmatic solution.

## Conclusion

The IMPACT research program was based on a partnership approach to developing locally relevant innovations that would improve access to primary healthcare for vulnerable populations. As such, differences were built into the program from the outset. Rather than allowing these differences to lead to separate unrelated reports of research findings, taking a theory-based approach to evaluating the interventions supported a more coordinated consideration of the ways in which access to primary healthcare can be improved. We observed that interventions that engaged consumers directly led to increases in their abilities to access healthcare, and this was particularly so when characteristics of providers and services were enhanced. The process of using a program logic approach was not designed to lead to a final definitive model; it is intended to contribute to ongoing thinking about access to primary healthcare. This paper provides an example of evaluating interventions to create change in complex systems with multiple actors and different pathways of influence. The approach taken may be useful for ongoing research design, implementation, evaluation and synthesis of findings.

## Data Availability

The datasets analyzed during the current study are not publicly available because participant consent was not obtained for public access. Data are available via the corresponding author on reasonable request.

## Abbreviations

IMPACT: Innovative Models Promoting Access-to-Care Transformation
LIP: Local Innovation Partnership
NSW: New South Wales
PC: Primary care
PHC: Primary health care
PMC: Primary medical care
PN: Practice nurse
GP: General practitioner
FP: Family physician
T2D: Type 2 Diabetes
